# Yield estimation of winter wheat in the Huang-Huai-Hai region using MODIS and meteorological data: spatio-temporal analysis and county-level modeling

**DOI:** 10.3389/fpls.2025.1721972

**Published:** 2025-12-15

**Authors:** Zhaoxia Lou, Deng Sun

**Affiliations:** 1 College of Agricultural Engineering, Shanxi Agricultural University, Jinzhong, China; 2 College of Engineering, Northeast Agricultural University, Harbin, China

**Keywords:** winter wheat, yield estimation, MODIS remote sensing, meteorological factors, machine learning, SHAP analysis

## Abstract

The Huang-Huai-Hai region is a major winter wheat production area in China. Achieving accurate yield estimation through high spatio-temporal resolution MODIS remote sensing combined with meteorological monitoring has become an important issue for ensuring food security. This study integrates multi-source MODIS satellite data (surface reflectance, leaf area index LAI, and fraction of absorbed photosynthetically active radiation FPAR) with precipitation and temperature data to construct a county-level winter wheat yield prediction model. First, spatio-temporal analyses were conducted on multidimensional parameters during key periods (overwintering, growth, and maturation). The results showed that reflectance responded sensitively to phenological changes; FPAR and LAI revealed photosynthetic capacity and canopy structure evolution; monthly mean precipitation and temperature exhibited significant spatio-temporal heterogeneity, providing data support for effective yield prediction. Next, PLS, RF, and BP models were constructed for the three periods. The BP model performed best across multiple periods, achieving the highest accuracy in the growth period (R²=0.81, RMSE=414.48 kg/ha) and was thus selected as the optimal window period and model. Shapley Additive Explanations (SHAP) analysis revealed the influence of model features on yield prediction, with specific reflectance bands, precipitation, and LAI identified as key contributing factors. Furthermore, the BP model was validated using remote sensing and meteorological data from the 2023 growth period, combined with county-level yields. The results showed R²=0.73 and RMSE=509.30 kg/ha, further confirming the model’s prediction accuracy and stability in practical applications. This study enables county-level estimation of winter wheat yield, providing scientific evidence and methodological reference for agricultural monitoring and food security assurance.

## 1 Introduction

As China’s main winter wheat producing area, the Huang-Huai-Hai region occupies an important position in terms of planting area and production volume nationwide (Ren et al., 2021; Zhao et al., 2023). The winter wheat yield in this region not only serves as a key pillar of China’s grain supply but also exerts a significant influence on the global food market. Despite its abundant soil resources, the winter wheat yield in this region exhibits pronounced spatial and temporal heterogeneity due to factors such as climate change, soil degradation, and uneven agricultural management. This instability has become increasingly severe with the rising frequency of extreme weather events, posing a direct threat to both the security of food production. Therefore, accurate crop yield prediction is of critical importance for ensuring the sustainability of regional food production. Traditional yield assessment methods typically rely on manual field surveys, which can provide accurate results (Lyu et al., 2024). However, their long data collection cycles, limited spatial coverage, and high costs make it difficult to meet the demands of large-scale, multi-scale yield estimation. Consequently, the timeliness and adaptability of traditional methods are no longer sufficient for the needs of modern agriculture.

With the rapid development of remote sensing technology, satellite-based crop yield estimation has gradually become a research hotspot (Ghazal et al., 2024; Shawon et al., 2025). The MODIS sensors carried by the Terra and Aqua satellite platforms offer high temporal resolution and multispectral observation capabilities (Sakamoto, 2020; Seguini et al., 2024). Their products, such as surface reflectance, leaf area index (LAI), and fraction of absorbed photosynthetically active radiation (FPAR), provide abundant data support for monitoring winter wheat growth and modeling yield (Unnisa et al., 2025; Zhang et al., 2025). At the same time, meteorological factors such as precipitation and temperature directly influence crop growth and are key information that cannot be ignored in yield prediction (Bhat et al., 2024; Chergui, 2022; Pede et al., 2019; Xu et al., 2019). Therefore, fusing satellite remote sensing with meteorological data provides an important pathway to improve the accuracy and stability of winter wheat yield estimation.

In recent years, in terms of modeling approaches, various machine learning (ML) methods have been applied to develop remote sensing–based yield prediction models, such as partial least squares regression (PLS), random forest (RF), and neural networks (Bai et al., 2021; Cai et al., 2019; Cao et al., 2020; Rufaioğlu et al., 2025; Tesfaye et al., 2021; Torgbor et al., 2023). Although these methods have demonstrated certain advantages in previous studies, their suitability varies in terms of handling different input data types, feature dimensions, and levels of nonlinearity. Thus, which specific modeling approach is most suitable for winter wheat scenarios still requires further investigation. Moreover, although machine learning models can achieve high predictive accuracy, some machine learning models exhibit “black-box” characteristics, making their internal mechanisms difficult to interpret and thus limiting a deeper understanding of how key variables contribute to the yield formation process. Winter wheat exhibits significant differences in its responses to external environmental conditions at different growth stages, which means that the predictive effect for yield may vary depending on the data acquisition period (Liu et al., 2022; Ovando et al., 2025). Therefore, effectively identifying the growth stages most critical to yield prediction and gaining an in-depth understanding of the mechanisms by which key variables influence yield prediction are of great importance for improving model accuracy and promoting the application of these techniques.

At present, existing studies have used satellite remote sensing data to estimate crop yields at different scales, such as national, provincial, or city levels, and have achieved substantial results (Lu et al., 2025; Medina et al., 2021; Seguini et al., 2024). However, research at the county scale remains relatively limited. As the basic unit for agricultural management and policy implementation, county-level yield estimates have direct guiding value for local agricultural production planning, resource allocation, and the implementation of precision agriculture. In comparison, county-level estimation not only requires higher predictive accuracy but also places greater demands on model stability and adaptability.

Based on the above background, this study focuses on the Huang-Huai-Hai region and aims to predict winter wheat yield at the county scale by integrating remote sensing parameters provided by MODIS, such as multispectral reflectance, LAI, and FPAR, with key meteorological variables including precipitation and temperature. The study conducts an in-depth spatio-temporal analysis of multidimensional parameters, with particular focus on three critical growth stages of winter wheat: overwintering, growth, and maturity. For these stages, three prediction models—PLS, RF, and BP(Back Propagation Neural Network)—are constructed to evaluate and compare their accuracy and adaptability in predicting winter wheat yield across different temporal windows. Furthermore, the Shapley Additive Explanations (SHAP) method is employed to provide a visual interpretation of the models, revealing the influence mechanisms of various parameters on yield prediction, and to further validate the predictive accuracy and stability of the models in practical applications. The main contributions of this study are as follows: by integrating multi-source remote sensing and meteorological data and leveraging machine learning methods, it identifies the optimal model and the most appropriate timing for winter wheat yield prediction, and demonstrates their application in practical scenarios. Through SHAP analysis, the interpretability and transparency of the models are enhanced, overcoming the “black-box” limitations observed in previous studies. This research not only offers a solution for high-precision county-scale winter wheat yield estimation but also provides a scientific basis and practical reference for agricultural monitoring and food security assurance.

The remainder of this paper is organized as follows: Section 2 introduces the study region, data acquisition, and the models used; Section 3 presents the spatiotemporal distribution of the data and the results of the model experiments; Section 4 explores the remote sensing and meteorological characteristics during the growth stages of winter wheat and model performance, and reveals the mechanisms of key factors and their influence on yield prediction; finally, Section 5 provides the conclusions of this study, its limitations, and directions for future research.

## 2 Materials and methods

### 2.1 Study area

**Figure 1:**
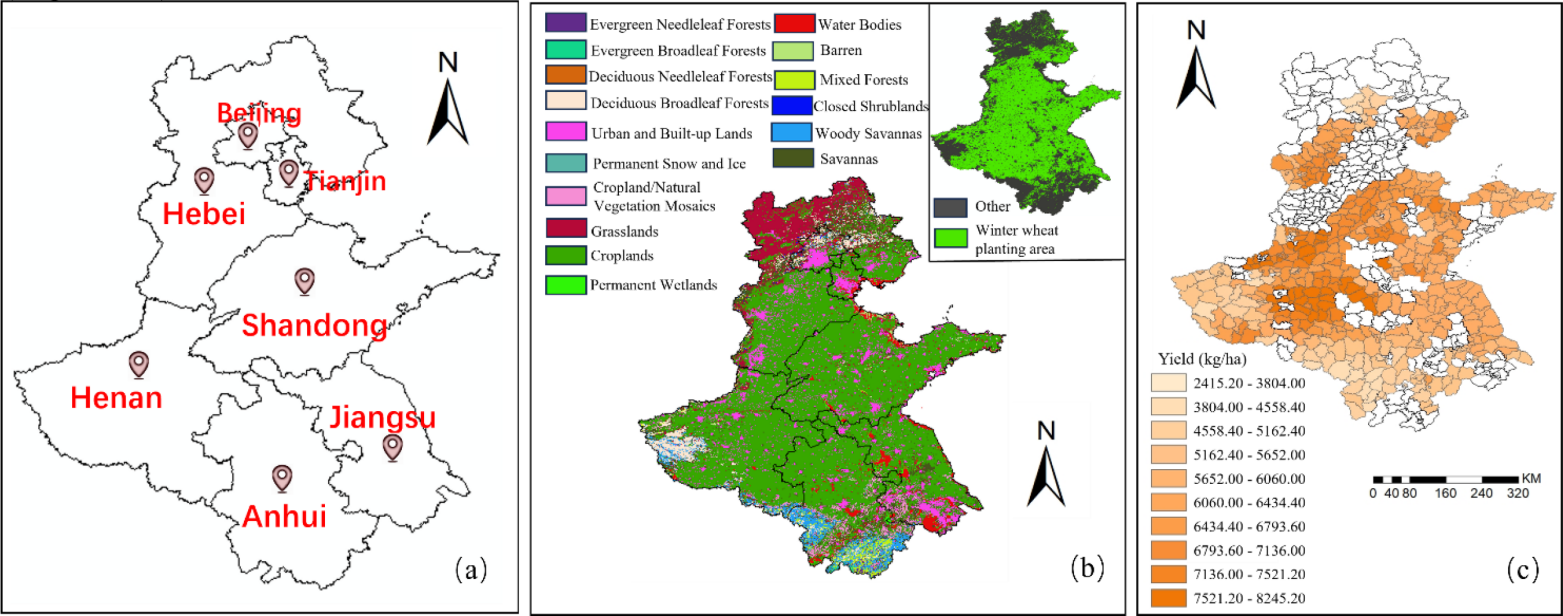
Geographic and agricultural information of the Huang-Huai-Hai region. **(a)** Geographic extent map of the Huang-Huai-Hai region; **(b)** Vegetation types and winter wheat planting areas in the Huang-Huai-Hai region; **(c)** Distribution of winter wheat yield in the Huang-Huai-Hai region in 2022.

The Huang-Huai-Hai region is located in eastern coastal China, spanning longitudes 110°21′–122°43′E and latitudes 29°41′–42°37′N (Li et al., 2024). It covers five major agricultural provinces—Anhui, Hebei, Henan, Jiangsu, and Shandong—as well as the two municipalities of Beijing and Tianjin (**Figure 1a**).

The region features a typical temperate continental monsoon climate, with an annual average temperature ranging from 8°C to 15°C and an annual average precipitation of approximately 500–900 mm (Ren et al., 2021). Characterized by flat terrain, favorable climatic conditions, and fertile soils, the Huang-Huai-Hai region is one of China’s most important grain-producing areas, particularly serving as the core area for winter wheat cultivation, which is sown in mid-October and harvested in June of the following year (Ren et al., 2021).

### 2.2 Data sources

#### 2.2.1 Time series data

Through the study of winter wheat, its entire growth cycle can be divided into three key phenological stages: the overwintering period, the growing period, and the maturity period (**Table 1**). The overwintering period is the first phase that winter wheat enters after sowing, usually from late December to mid-February of the following year, during which winter wheat remains dormant. The growing period is the main development phase of winter wheat, starting in mid-March, during which plant growth accelerates and the crop enters key phases such as regreening, jointing, and heading. The maturity period begins in early May, when plant growth ceases, and the grains undergo filling and gradually ripen (Wu et al., 2023). Based on the timing of the three phenological stages of winter wheat and the needs of the study, remote sensing data covering the study area for 2022 and 2023 were selected.

**Table 1:** Data acquisition timing for 2022 and 2023 (excluding the 2021 period).

Phenological period	Time range	Time selection		
		Reflectance (Ref1-7)	LAI and FPAR	Precipitation and temperature
Overwintering period	December 20-February 15	January 31	February 2	January
Growth period	March 15-May 1	March 31	March 30	March
Maturity period	May 1-June 15	May 12	May 13	May

The data acquisition timing for 2022 and 2023 is consistent. At the time points of MODIS satellite data acquisition, winter wheat was in the overwintering, jointing, and filling to maturity condition.

To ensure high spatiotemporal continuity and reliability of the vegetation functional features, spectral features, and meteorological information required for winter wheat yield prediction, this study selected MODIS satellite remote sensing products and high-resolution national meteorological data. The satellite data were obtained from the Earth Observing System Data and Information System (EOSDIS) of the National Aeronautics and Space Administration (NASA). The MYD09GA (Level 2G), provided by the MODIS sensor aboard the Aqua satellite, is a daily surface reflectance product with a resolution of 500 m after atmospheric correction, covering seven bands including blue, green, red, near-infrared, and shortwave infrared (Ref1–7). These spectral bands are highly sensitive to key crop growth indicators, enabling accurate characterization of the spectral variations of winter wheat across different growth stages. The MCD15A3H (Level 4) product generates four-day composite images of LAI and FPAR at 500 m resolution. These indicators are obtained through inversion of a radiation transfer model and can accurately describe the growth dynamics and photosynthetic capacity of winter wheat, providing essential vegetation functional features for yield prediction (Román et al., 2024). MODIS products feature high temporal resolution, long-term sequence stability, and extensive spatial coverage. They are accompanied by quality assurance (QA) files, which enable effective screening and removal of cloud-contaminated and low-quality pixels, providing a reliable data foundation for subsequent regional modeling (Román et al., 2024). Meteorological data were obtained from the National Tibetan Plateau/Third Pole Environment Data Center. The data includes monthly precipitation and monthly mean temperature for China in 2022–2023 at a 1 km spatial resolution, with precipitation expressed in 0.1 mm units and mean temperature in 0.1°C units. This dataset was generated for China using a Delta spatial downscaling approach based on the global 0.5°climate dataset released by CRU and the high-resolution global climate dataset released by WorldClim. It has high accuracy and spatial detail and is suitable for regional-scale yield simulation (Peng, 2025a, b; Peng et al., 2019).

Observational data were selected based on three considerations: crop phenological stage characteristics, image quality, and temporal and spatial consistency. Overwintering period— in late January, winter wheat is in a stable overwintering stage, during which vegetation spectral signals are low and the background is stable, clearly reflecting the post-winter canopy condition. Growth period — in late March, during the jointing stage, winter wheat undergoes rapid growth and exhibits the most sensitive spectral variations, which helps capture key physiological information of the growing period. Maturity period— in early May, during the grain-filling period, the canopy structure and water status exhibit the greatest differences as the crop approaches maturity, and the spectral characteristics provide significant indicators for yield formation. Furthermore, to ensure complete spatial coverage and pixel quality, available images within the time range were screened for quality, selecting observation dates with minimal cloud cover and the highest proportion of valid pixels, thereby ensuring spatial continuity and data reliability. At the same time, the acquisition dates for all data features were kept consistent to minimize phenological variation caused by temporal differences and to maintain consistency of multi-source data inputs for the models. Finally, the selected dates provide effective reflectance information covering most counties of the study area, avoiding biases caused by regional data gaps. The specific observation dates are shown in **Table 1**. These dates not only represent the typical growth status of winter wheat at different stages but also maximize the use of remote sensing information under controlled data quality, providing reliable input data for yield model construction.

#### 2.2.2 Winter wheat cultivation area

According to the winter wheat planting area statistics provided by the National Bureau of Statistics, China’s winter wheat planting area has remained relatively stable over the past decade, with little fluctuation. To extract the winter wheat planting region, this study used the MCD12Q1 product to obtain 2022 land cover type data. This product, based on the International Geosphere-Biosphere Programme (IGBP) land cover classification system, divides the Earth’s surface into 17 typical vegetation types (Ma and Liang, 2022). Given that cropland resources in the Huang–Huai–Hai region are primarily used for the winter wheat-summer maize rotation system, with winter wheat accounting for more than 90% of cropland coverage, the “Croplands” category in the IGBP classification system was selected as the winter wheat planting region for this study (Wang et al., 2024). By overlaying this land cover type data with the administrative boundary vector map of the study area and performing spatial clipping, the distribution of winter wheat cultivation within the study area was extracted (**Figure 1b**) (Guo et al., 2024). The satellite remote sensing and meteorological data were then clipped to the winter wheat cultivation area, and the mean values within each county were extracted using the county-level vector map, serving as input data for the subsequent modeling.

#### 2.2.3 Crop yield data

The Huang–Huai–Hai region encompasses seven administrative units: Beijing, Tianjin, Hebei, Henan, Shandong, Anhui, and Jiangsu, comprising a total of 696 county-level administrative units. The winter wheat yield data used in this study come from county-level statistics in the statistical yearbooks published by local statistical bureaus for 2022 and 2023 (yield in 2022 is shown in **Figure 1c**). To achieve spatial matching between the remote sensing and meteorological data and the actual yields, the yield data were mapped to the corresponding county-level administrative units, ensuring spatial consistency between the statistical data and the analysis units. However, due to some counties not publicly releasing winter wheat yield data for 2022 and 2023 or having missing/unavailable data, only 780 county-level yield (449 in 2022, 331 in 2023) records were successfully collected and used as valid samples for model training and validation in this study.

### 2.3 Winter wheat yield prediction model

To achieve accurate county-scale estimation and comparative analysis of winter wheat yield, this study constructed three representative modeling approaches: PLS, RF, and BP. PLS is a multivariate linear regression method that extracts latent variables with maximum covariance with the response variable, retaining the main information relevant to the response variable to establish the regression relationship. In this study, 8 principal components were extracted from the input variables, with a cumulative contribution rate exceeding 90%, effectively preserving the main information of the independent variables. RF is a non-parametric model based on the ensemble learning concept, which constructs multiple decision trees on random subsets of the data and combines the predictions of all trees using a weighted average. In this study, the RF model was configured with the number of decision trees (NumTrees) set to 100 and the minimum number of leaf node observations (MinLeafSize) set to 5. The BP neural network adopts a multi-hidden-layer structure, learning the complex relationship between input and target variables through layer-by-layer nonlinear mapping, exhibiting strong function approximation and pattern recognition abilities. In this study, the BP model was configured with a hidden layer size (HiddenSizes) of 25, a target number of epochs (Target Epoch) of 1000, and weight updates were performed using the Bayesian regularization backpropagation algorithm (trainbr) to enhance the model’s generalization ability and reduce the risk of overfitting.

The input variables for the models in this study included MODIS satellite remote sensing data (seven-band surface reflectance, LAI, and FPAR) and meteorological data (monthly precipitation and temperature), totaling 11 input features. These feature variables were used to construct yield models for three phenological stages of winter wheat: overwintering, growing, and maturity periods. All models were trained and tested using 2022 data, with the dataset split into training and testing sets at an 8:2 ratio. Five-fold cross-validation was applied to enhance model robustness and generalization capability.

During model evaluation, the root mean square error (RMSE) and the coefficient of determination (R²) were calculated using Equations 1 and 2 to assess the predictive performance of the models. The model was implemented by using MATLAB R2024a on an Intel Core i5-10300H CPU, 16 GB of RAM, and an NVIDIA GeForce RTX 1650 GPU.

(1) $$RMSE=\sqrt{\frac{\sum_{i=1}^{N}{\left(y_{i}-y_{i}^{\prime }\right)}^{2}}{N}}$$

(2) $$R^{2}=\sqrt{\frac{\sum_{i=1}^{N}{\left(y_{i}-\bar{y}\right)}^{2}-\sum_{i=1}^{N}{\left(y_{i}-y_{i}^{\prime }\right)}^{2}}{\sum_{i=1}^{N}{\left(y_{i}-\bar{y}\right)}^{2}}}$$

N being the sample size, $y_{i}$ the measured value, $y_{i}^{\prime }$ the predicted values, and $\bar{y}$ the mean of the values.

After completing model training and evaluation, the optimal model–phenological stage combination was selected based on the assessment metrics as the final configuration for regional yield prediction. Subsequently, the optimal model and the 2023 remote sensing and meteorological data corresponding to the selected phenological stage were used to estimate and simulate winter wheat yield for 2023. The results were compared with actual county-level statistics for 2023 to assess the model’s predictive accuracy and stability in practical applications, thereby validating its suitability and potential for wider application in winter wheat yield estimation.

## 3 Results and analysis

### 3.1 Spatio-temporal analysis of data

#### 3.1.1 Winter wheat yield data analysis

**Figure 2:**
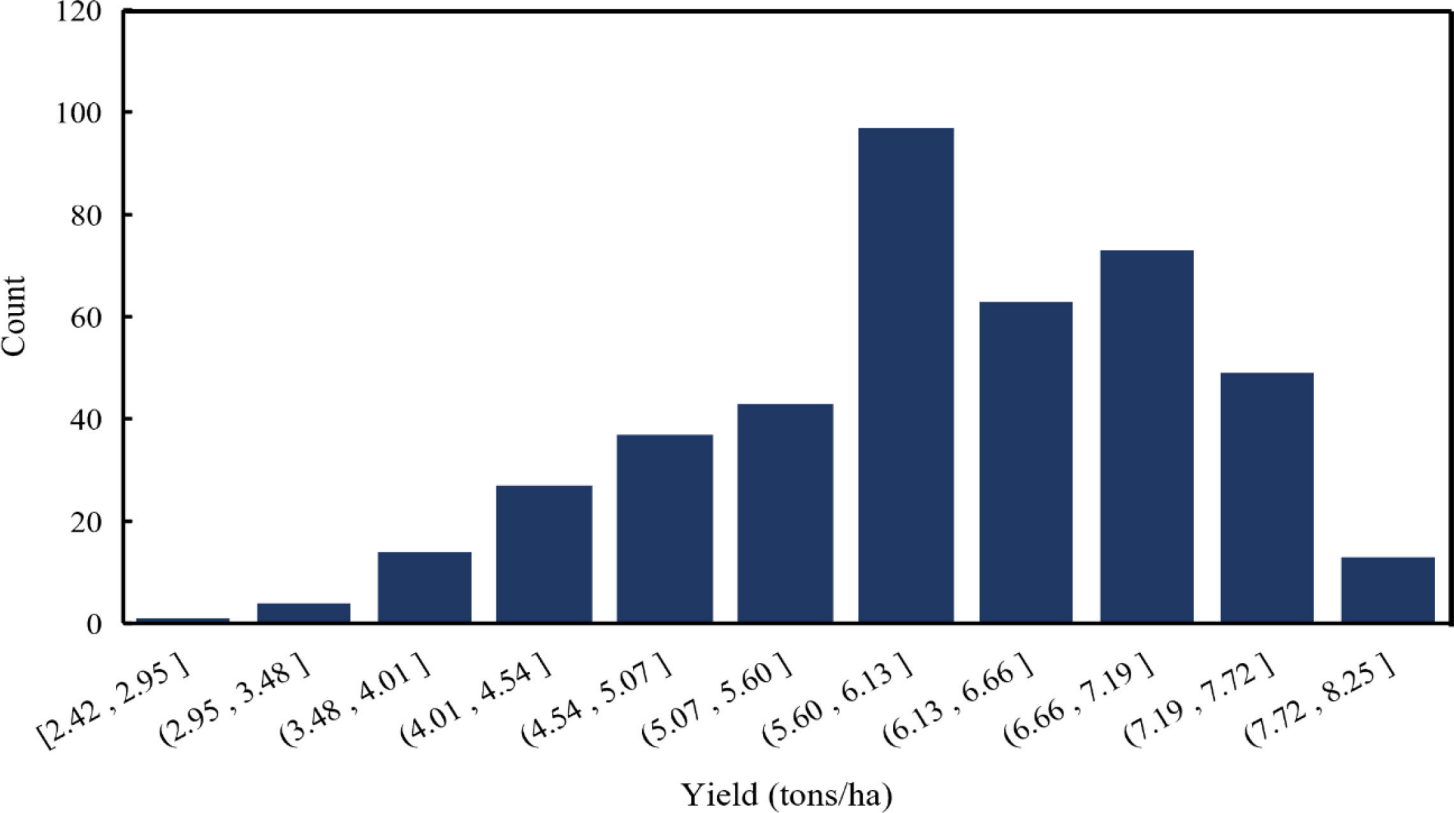
Yield frequency distribution at the county scale in the Huang–Huai–Hai region in 2022.

According to the 2022 statistical data released by the National Bureau of Statistics, significant differences in winter wheat yield were observed across counties in the Huang–Huai–Hai region (**Figure 1c**), with a minimum of 2415 kg/ha and a maximum of 8245 kg/ha, showing pronounced spatial imbalance. The yield frequency distribution (**Figure 2**) shows that most counties have yields ranging from 5.50 to 7.20 tons/ha, with only a few falling below 3.50 tons/ha. This suggests that the overall yield level of winter wheat in this region is relatively high, while noticeable regional disparities still exist. From a spatial distribution perspective (**Figure 1c**), high-yield areas are mainly concentrated in eastern Henan and western Shandong. These regions benefit from flat terrain, favorable climatic conditions, fertile soils, and well-developed farming systems, which collectively contribute to high productivity. In contrast, winter wheat yields in western Henan and central Anhui are relatively low. According to reports from the meteorological bureau, this may be attributed to soil moisture deficits resulting in insufficient soil water availability; extreme weather events such as persistent hot, dry winds and continuous rainfall during the plum rain season, which adversely affect grain filling; and poor soil fertility, with problems such as soil compaction or severe salinization (Li et al., 2025). Overall, the spatial heterogeneity of winter wheat yields at the regional scale provides a solid experimental basis for remote sensing–based yield estimation modeling.

#### 3.1.2 Analysis of spectral reflectance data

**Figure 3:**
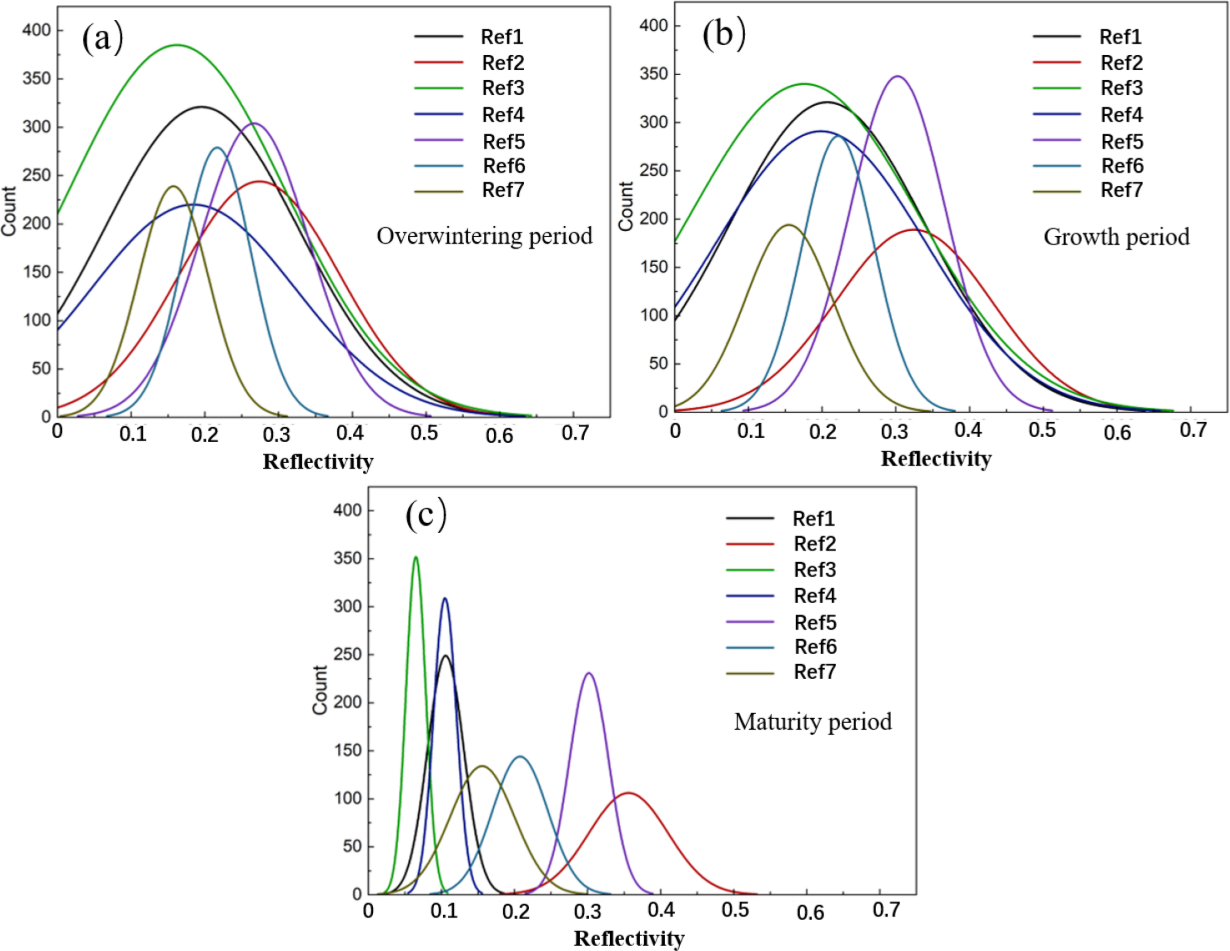
Frequency fitting curves (Gaussian) of spectral reflectance (7 bands, Ref1-7) at different stages. **(a)** Overwintering period; **(b)** Growth period; **(c)** Maturity period.

The frequency fitting curves of the 7-band spectral reflectance obtained from the MYD09GA product are shown in **Figure 3**. The reflectance distributions of the visible bands Ref1, Ref3, Ref4 and the near-infrared band Ref2 range from 0 to 0.7 during the overwintering and growing stages. However, in the maturity stage, the reflectance ranges of Ref1, Ref3, and Ref4 contract to 0-0.2, while Ref2 narrows to 0.2-0.5. In addition, the frequency peaks of Ref1, Ref3, and Ref4 remain relatively stable during the overwintering and growing stages but become sharper and more concentrated in the maturity stage, indicating that the winter wheat canopy tends to be consistent at this stage, with similar spectral characteristics. For the shortwave infrared band Ref5, reflectance is distributed within 0-0.5 during the overwintering and growing stages, but significantly contracts to 0.2-0.4 in the maturity stage, accompanied by a decrease in frequency peak. The shortwave infrared bands Ref6 and Ref7 maintain relatively stable reflectance ranges of 0-0.4 in the three stages, yet their frequency peaks decline notably in the maturity stage, with Ref6 showing a more pronounced reduction.

#### 3.1.3 Spatiotemporal analysis of climate factors

**Figure 4:**
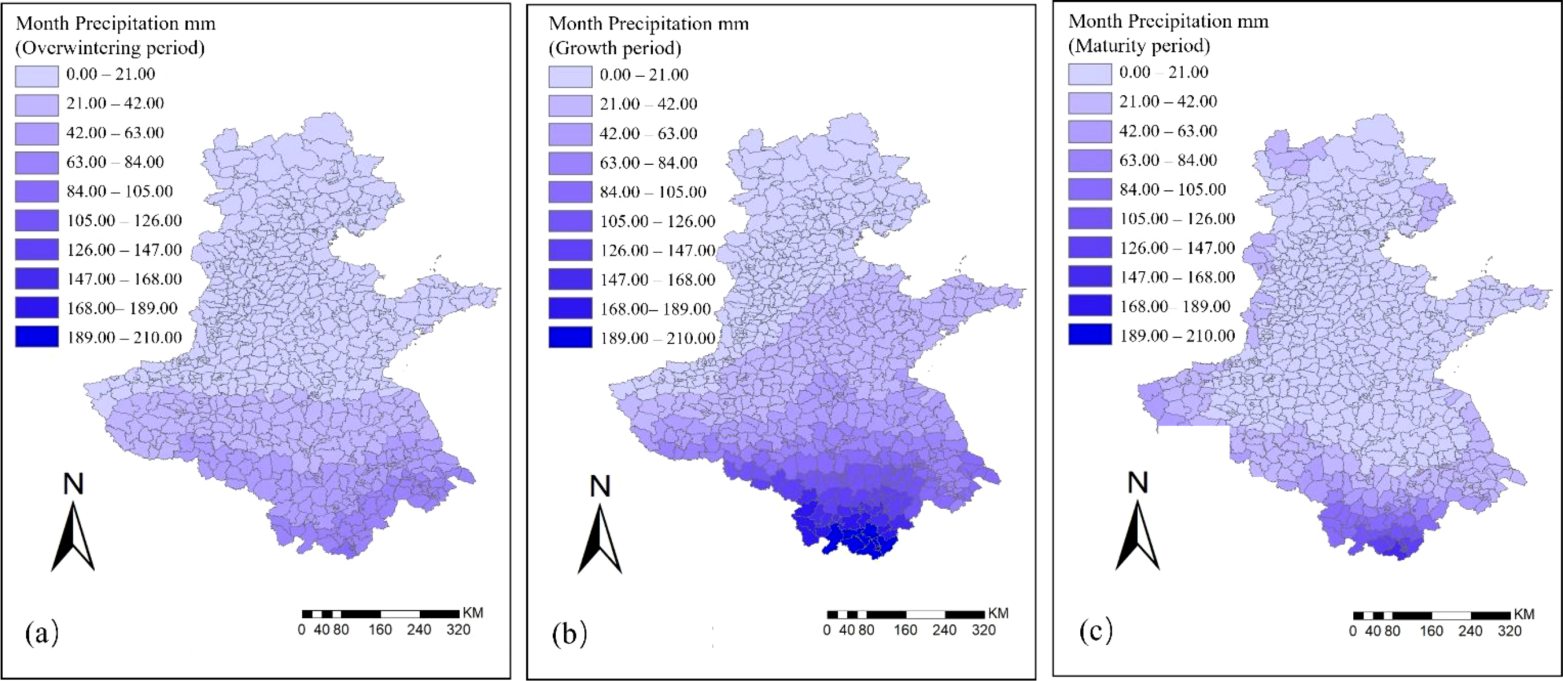
Spatial and temporal distribution of monthly precipitation in the Huang–Huai–Hai region in 2022. **(a)** Overwintering period; **(b)** Growth period; **(c)** Maturity period.

During the growth of winter wheat, precipitation and temperature, as important climatic factors, have a significant influence on its growth and development. The spatial and temporal distribution of monthly precipitation for winter wheat in the Huang-Huai-Hai region during the overwintering, growing, and maturity periods is shown in **Figure 4**.

During the overwintering period, the spatially averaged monthly precipitation across the Huang-Huai-Hai region was 22.5 mm, with about 60% of the counties receiving 0–21 mm. In particular, Beijing, Tianjin, Hebei, and Shandong experienced relatively low rainfall, showing typical characteristics of a winter drought climate. Precipitation in southern Henan, Anhui, and parts of Jiangsu was slightly higher but did not exceed 90 mm, indicating that water input during this stage was generally limited, and winter wheat relied on antecedent soil moisture to sustain growth (Chen et al., 2024).

During the growing period, the spatially averaged monthly precipitation reached 46 mm, with Shandong, Jiangsu, Henan, and Anhui experiencing a significant increase in rainfall, providing favorable water conditions for the rapid growth of winter wheat. A high-rainfall zone of 147–210 mm formed in parts of southern Anhui. However, Beijing, Tianjin, and Hebei still maintained low precipitation levels of 0–21 mm, which may have imposed water stress on crop growth in these areas.

During the maturity period, the spatially averaged monthly precipitation decreased to 21 mm, with rainfall in Shandong, Jiangsu, Henan, and Anhui significantly reduced. About 70% of the counties recorded precipitation in the range of 0–21 mm, with relatively higher rainfall of 105–168 mm observed only in parts of southern Anhui.

**Figure 5:**
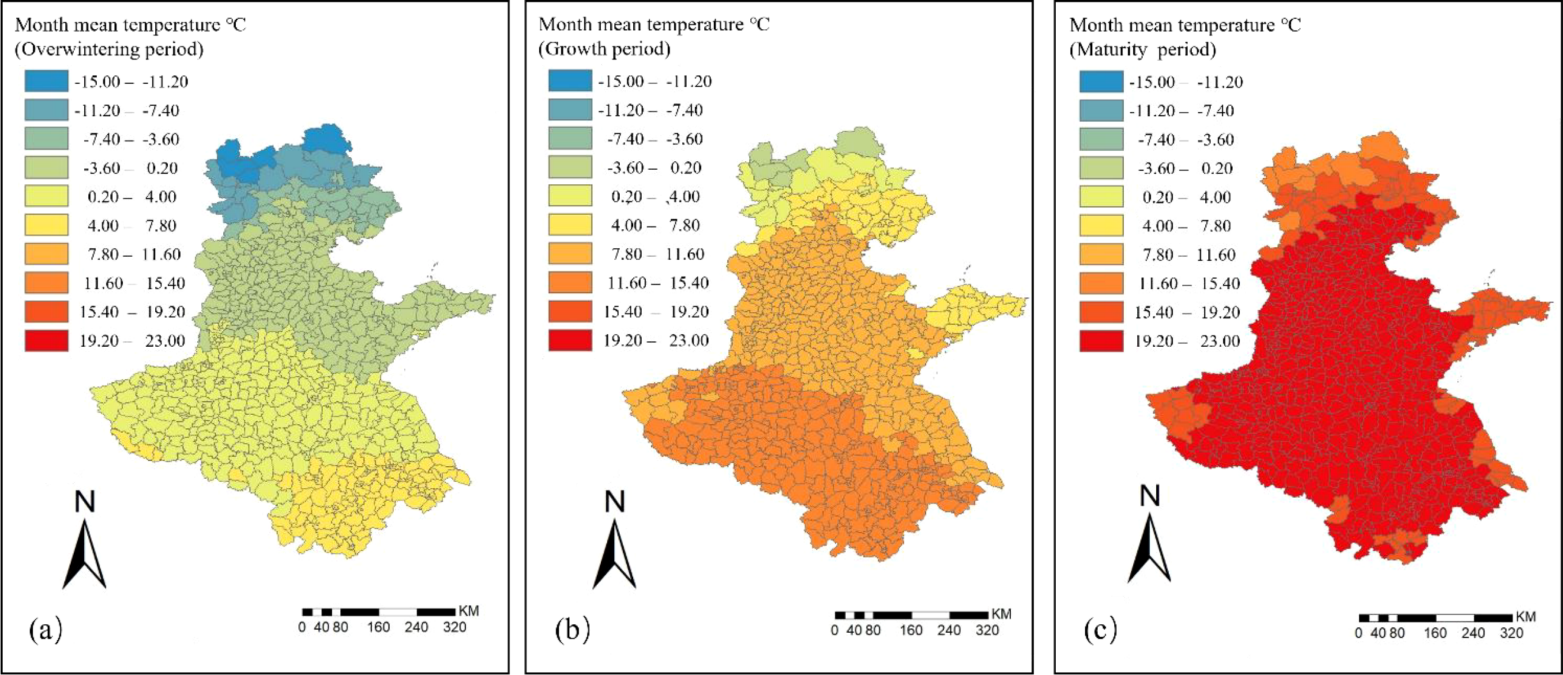
Spatial and temporal distribution of monthly mean temperature in the Huang–Huai–Hai region in 2022. **(a)** Overwintering period; **(b)** Growth period; **(c)** Maturity period.

The spatial and temporal distribution of monthly average temperatures for winter wheat in the Huang-Huai-Hai region during the overwintering, growing, and maturity periods is shown in **Figure 5**. During the overwintering period, temperatures across the Huang-Huai-Hai region were generally low, with the spatially averaged of monthly mean temperature at 1°C. About 40% of the counties experienced monthly average temperatures below 0°C, mainly in Beijing, Tianjin, Hebei, and most parts of Shandong. Northern Hebei had the lowest temperatures, with some local areas dropping to –10°C. In contrast, temperatures in Henan, Anhui, and Jiangsu were relatively higher, but did not exceed 7°C.

During the growing period, the spatially averaged of monthly mean temperature rose to 10°C. About 45% of the counties had monthly average temperatures above 11°C, mainly concentrated in most parts of Anhui and Henan, with the highest temperatures reaching 15°C. In most areas of Hebei, Shandong, and Jiangsu, monthly average temperatures ranged from 7.8°C to 11.6°C, with only five counties in northern Hebei experiencing temperatures below 0°C.

During the maturity period, temperatures increased further, with the spatially averaged of monthly mean temperature reaching 20°C. About 90% of the counties had monthly average temperatures between 19.2°C and 23°C. Some counties in northern Hebei, eastern Shandong, and western Henan experienced relatively lower temperatures, but the minimum remained above 13°C, indicating overall sufficient heat resources, which are favorable for grain filling and maturation.

#### 3.1.4 Spatiotemporal analysis of FPAR and LAI

**Figure 6:**
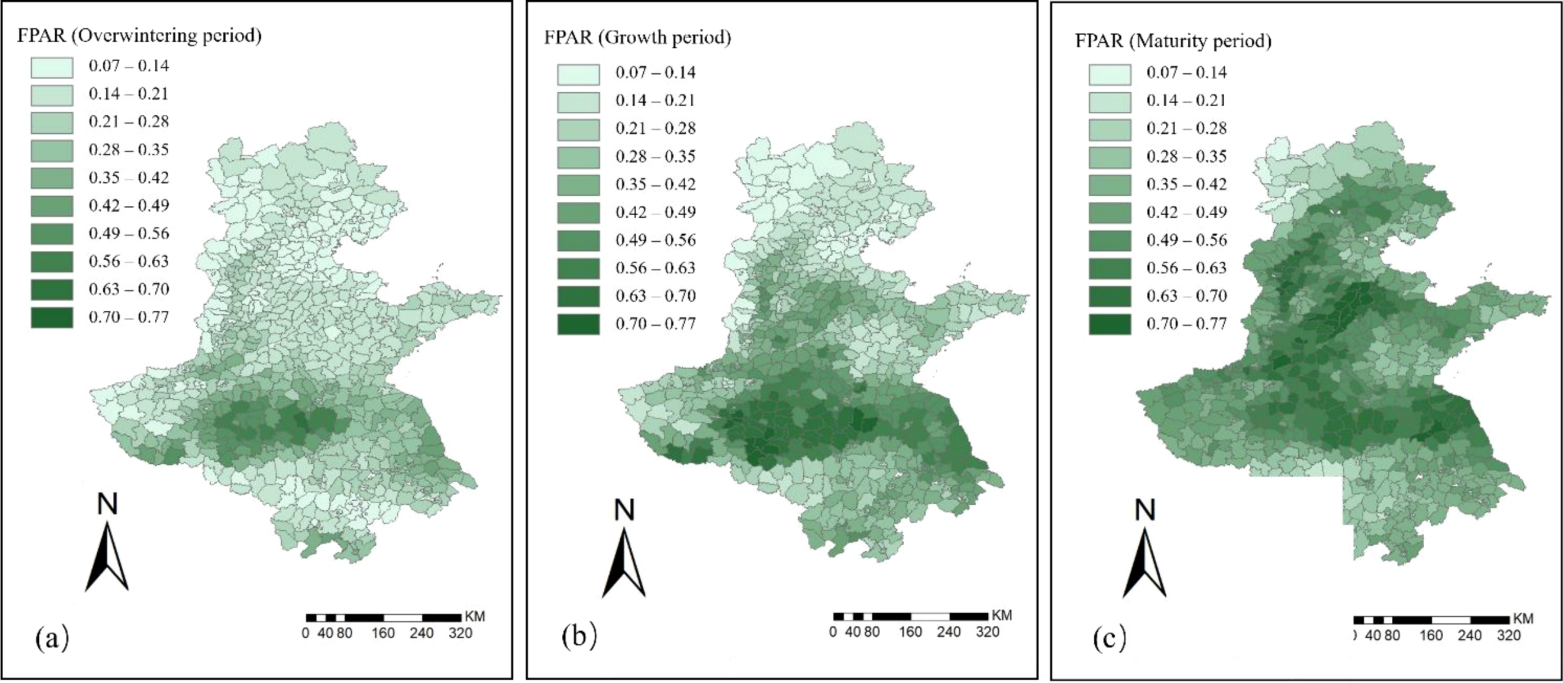
Spatiotemporal distribution of FPAR for winter wheat in the Huang–Huai–Hai region in 2022. **(a)** Overwintering period; **(b)** Growth period; **(c)** Maturity period.

The spatiotemporal distribution of FPAR for winter wheat in the Huang–Huai–Hai region across the three phenological stages is shown in **Figure 6**.

During the overwintering stage, the average FPAR of the region was 0.23, generally at a low level, indicating low light energy absorption efficiency of the crop canopy and restricted photosynthesis. In most areas, FPAR values were distributed between 0–0.28, while in eastern Henan and northern Anhui the values were relatively higher, reaching 0.50–0.63, indicating that crops in these regions had a certain advantage in light energy absorption and photosynthetic capacity.

After entering the growing stage, crops grew rapidly, and FPAR increased significantly, with the mean value rising to 0.35, reflecting a marked enhancement in the crop light energy absorption efficiency. Areas with high FPAR (>0.50) expanded rapidly from the scattered distribution in the overwintering stage, with a significant increase in coverage. However, Beijing, Tianjin, and northern Hebei still remained at a relatively low level of 0–0.28.

During the maturity stage, the mean of FPAR further increased to 0.47, and most areas had already entered a high FPAR (>0.50) status, indicating that the crop canopy had strong light energy absorption efficiency at this stage, with its spatial distribution showing high consistency.

**Figure 7:**
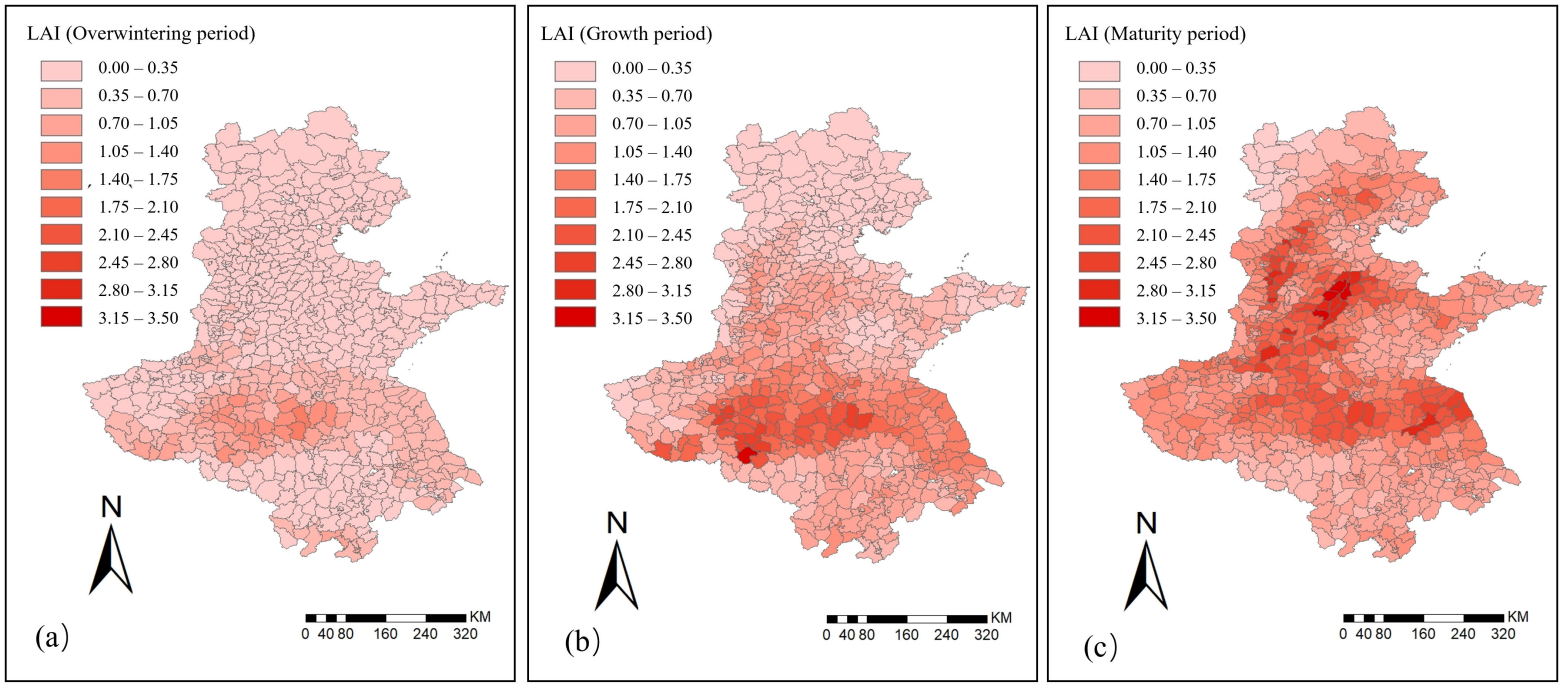
Spatiotemporal distribution of LAI for winter wheat in the Huang–Huai–Hai region in 2022. **(a)** Overwintering period; **(b)** Growth period; **(c)** Maturity period.

The spatiotemporal distribution of LAI for winter wheat in the Huang–Huai–Hai region during the three phenological stages is shown in **Figure 7**. During the overwintering stage, the average LAI of the region was only 0.33, which remained at a relatively low overall level. About 80% of the region had LAI values ranging from 0.0 to 0.7. Only in eastern Henan and northern Anhui was LAI relatively higher, but the maximum still did not exceed 1.5, indicating that canopy coverage was low at this stage.

During the growing stage, the average LAI of the region increased to 0.84. About 80% of the region had LAI values concentrated between 0.0 and 1.4, showing a significant increase in canopy coverage. Notably, contiguous high-value zones with LAI ranging from 2.1 to 3.5 formed in eastern Henan and northern Anhui. In contrast, although LAI increased in Beijing, Tianjin, Hebei, and Shandong, a large portion of counties in these areas remained at relatively low levels of 0–0.7.

During the maturity stage, canopy coverage of the crop reached its peak, with the average LAI of the region increasing to 1.37. About 75% of the region had LAI values ranging from 0.7 to 2.1. Notably, the high-value zones expanded from Henan and Anhui to parts of Hebei, Shandong, and Jiangsu. At this stage, the crop canopy was fully developed, and the green leaf area reached its maximum.

### 3.2 Performance comparison of different models in yield prediction

To comprehensively evaluate the performance differences of different modeling methods in winter wheat yield prediction, this study compared the predictive capabilities of the BP, RF, and PLS models across multiple growth stages, with the results summarized in **Table 2**.

**Table 2:** Performance evaluation of the three models across different phenological stages.

Period	Evaluation index	BP	RF	PLS
Overwintering period	R^2^	0.68	0.66	0.60
	RMSE	554.51	597.31	657.27
Growth period	R^2^	0.81	0.77	0.67
	RMSE	414.48	459.11	572.31
Maturity period	R^2^	0.74	0.69	0.62
	RMSE	491.15	538.29	632.57

Specifically, the BP model exhibited the best predictive performance during the growing period, with an R² of 0.81, significantly higher than that in the overwintering (0.68) and maturity periods (0.74). The RF model performed slightly worse than the BP model across all stages, with an R² of 0.77 in the growing period, still notably higher than in the overwintering (0.66) and maturity periods (0.69). In contrast, the PLS model showed lower predictive accuracy than both BP and RF, with an R² of only 0.67 during the growing period, and 0.60 and 0.62 in the overwintering and maturity periods, respectively, indicating a relatively limited ability to capture complex nonlinear relationships among variables.

**Figure 8:**
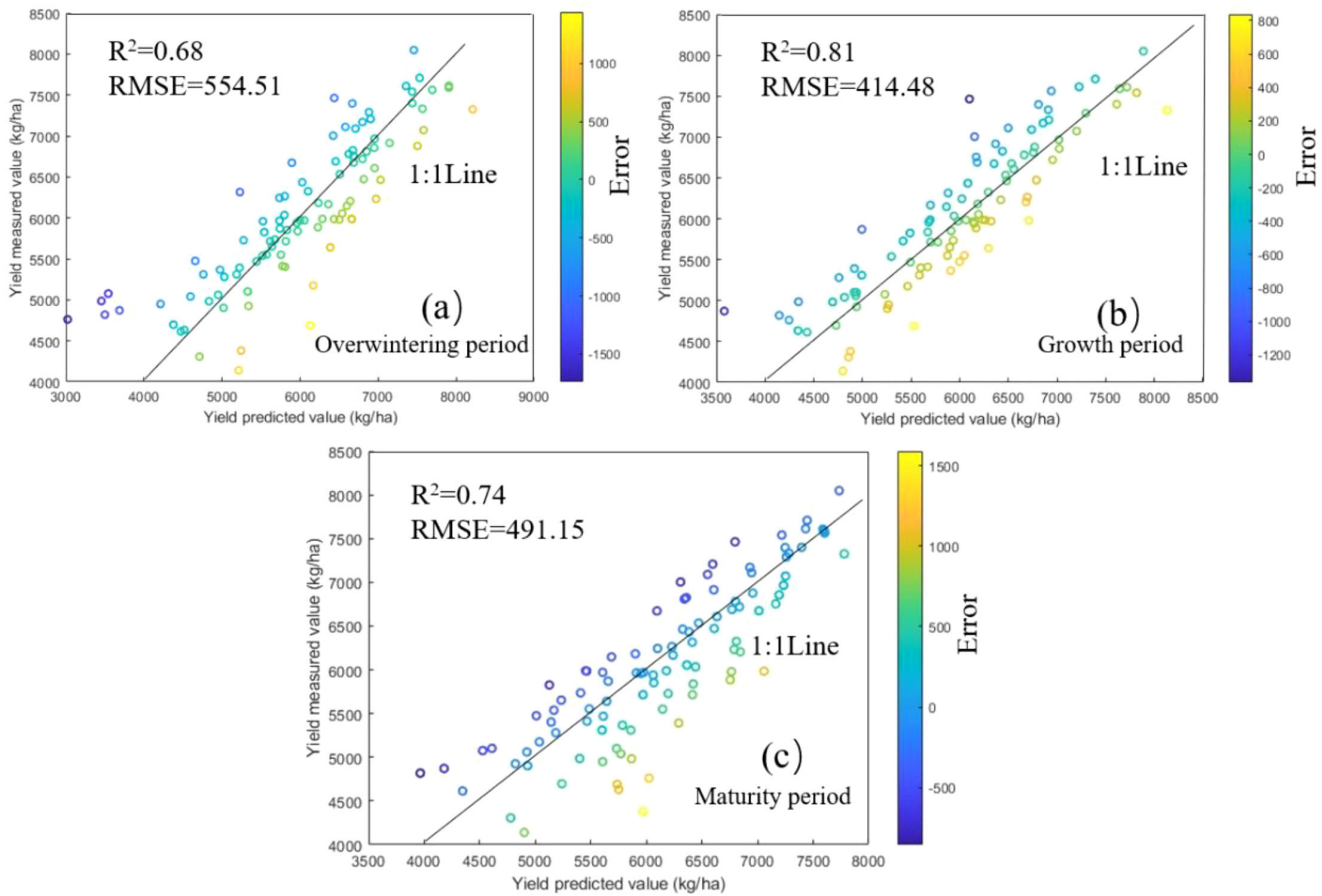
Prediction effect of the BP model across different periods. **(a)** Overwintering period; **(b)** Growth period; **(c)** Maturity period.

The prediction accuracy of the BP model was superior to that of the RF and PLS models across the three periods, and its yield prediction effect is shown in **Figure 8**. During the growth period, the BP model achieved the highest R² and the lowest RMSE (414.48 kg/ha), with the vast majority of test samples concentrated around the 1:1 line. In the overwintering period, R² was the lowest and RMSE the highest (554.51 kg/ha), with test samples being the most scattered, among which the yields in several low-yield regions (<5000 kg/ha) were severely underestimated or overestimated. In the maturity period, the R² reached 0.74 with an RMSE of 491.15 kg/ha, and test samples were evenly distributed around the 1:1 line, though yields in several low-yield regions (<5000 kg/ha) were seriously overestimated. Overall, the BP model demonstrated a clear advantage during the growth period when information is most abundant, highlighting its suitability for handling nonlinear relationships in crop yield prediction tasks.

### 3.3 Validation and application of the yield prediction model

Through the above analysis, the growth period was identified as the optimal window for winter wheat yield prediction, during which the BP model performed best. Based on this, the present study employed 11 variable parameters from the 2023 winter wheat growth period (seven multispectral reflectance Ref1–Ref7; FPAR and LAI; and monthly precipitation RAIN, and monthly mean temperature TEMP) as model inputs. Winter wheat yield was predicted using the established BP model, and the model was validated and applied against county-level yield data provided by the National Bureau of Statistics.

**Figure 9:**
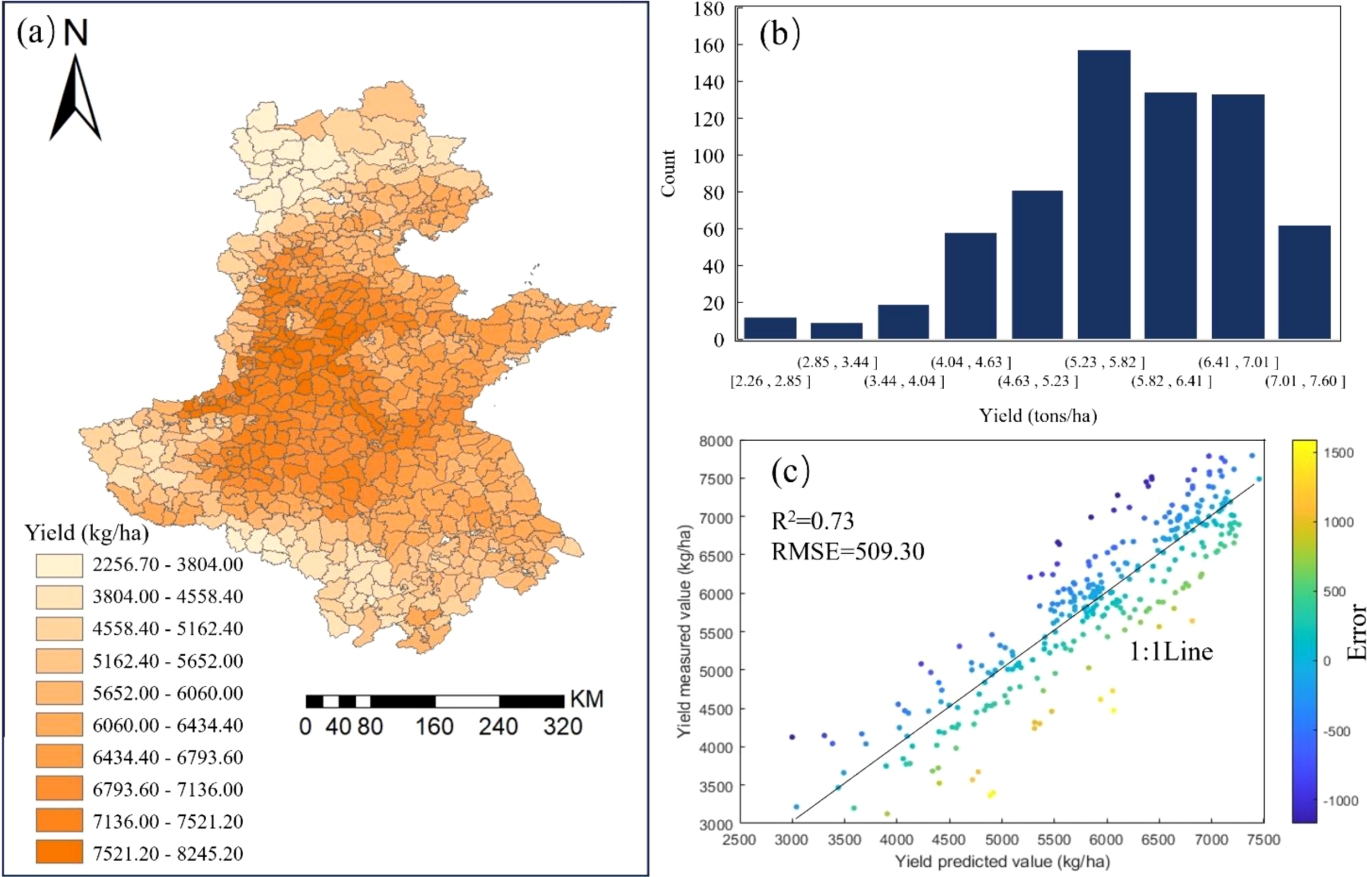
Validation results with 2023 data. **(a)** Spatial distribution of predicted yields; **(b)** Frequency distribution of predicted yields; **(c)** Prediction accuracy of yields.

The predicted distribution of winter wheat yield in the Huang-Huai-Hai region for 2023 is shown in **Figure 9a**. The southern part of Hebei, the eastern part of Henan, and the western part of Shandong are high-yield regions for winter wheat, while the northern part of Hebei, the western part of Henan, and the southern part of Anhui show relatively lower yields. The predicted winter wheat yield distribution in the Huang-Huai-Hai region for 2023 is somewhat similar to that of 2022. From the yield frequency distribution (**Figure 9b**), it can be seen that the minimum winter wheat yield is 2.26 tons/ha and the maximum is 7.60 tons/ha. Most counties have yields concentrated in the range of 5.20-7.00 tons/ha, while areas with yields in the range of 2.20-3.50 tons/ha are relatively few, indicating that the overall yield level of winter wheat in the region is relatively high, but certain regional differences still exist. Using the 2023 county-level yield data from the National Bureau of Statistics for validation, the winter wheat yield prediction results are shown in **Figure 9c**. The validation data yielded an R² of 0.73 and an RMSE of 509.30 kg/ha. The figure shows that data from low-yield areas are more dispersed, while high-yield areas are concentrated near the 1:1 line, indicating that the model is robust for high-yield scenarios but still requires further optimization for low-yield conditions. Overall, the BP neural network model based on the growth period can relatively accurately capture the spatial distribution characteristics of winter wheat yield in the Huang–Huai–Hai region, confirming its reliability and stability for county-scale yield prediction in practical applications.

## 4 Discussion

### 4.1 Change characteristics of vegetation and meteorological factors and their relationship with yield

Through the analysis of spectral reflectance frequency curves (**Figure 3**), winter wheat exhibits an overall trend of “ Spectral compression and peak migration” across the three growth stages, reflecting the phasic changes in spectral response to vegetation growth. These changes may be attributed to winter wheat entering the maturity stage, during which variations in canopy chlorophyll and water content lead to reduced reflectance, weakened inter-regional differences, and narrower fluctuation ranges (Shen et al., 2013; Yue et al., 2024). Meanwhile, in some bands, the reflectance of most winter wheat pixels within the region tends to cluster around a certain “high-frequency value,” resulting in a more concentrated and consistent overall distribution. Overall, the MYD09GA reflectance in each band shows a sensitive spectral response to phenological changes in winter wheat, providing a spectral basis for yield modeling at different stages.

Further analysis of climatic factors revealed (**Figures 4**, **5**) that during the growth of winter wheat, both monthly precipitation and monthly mean temperature exhibited pronounced temporal staging and spatial heterogeneity. In terms of precipitation, regional monthly precipitation was relatively low during the overwintering and maturity stages, showing strong spatial consistency. Although precipitation was limited in the overwintering stage, winter wheat reduced water consumption by entering dormancy, thereby ensuring the safe overwintering. In the maturity stage, moderate precipitation can meet the water requirements for grain filling, but excessive rainfall impairs root respiration and consequently affects grain filling (Nishio et al., 2024; Pais et al., 2023). By contrast, precipitation during the growing period increased significantly, with marked spatial variability in water conditions across different regions, reflecting a highly uneven distribution of rainfall. In terms of temperature, the monthly mean temperature during the overwintering and growing periods exhibited considerable spatial heterogeneity, with relatively lower temperatures in some northern areas constraining crop growth potential. In contrast, the spatial distribution of regional monthly mean temperature during the maturity stage showed high consistency, with relatively high and uniform temperatures overall, which were conducive to grain filling and maturation (Nishio et al., 2024). Overall, climatic factors played a critical role in the growth, development, and yield formation of winter wheat, while also providing an important data foundation for remote-sensing-based yield estimation models.

During the growth process of winter wheat, the dynamic variations of FPAR and LAI clearly illustrated the evolution of its photosynthetic capacity and growth rhythm (**Figures 6**, **7**). In the overwintering stage, constrained by low temperatures and limited precipitation, both FPAR and LAI remained at low levels. The stress of cold was particularly evident in Hebei and Shandong, where green leaf area was sparse and light absorption efficiency was low. In the growing stage, as temperatures rose significantly and precipitation conditions improved, the crop canopy expanded rapidly, with FPAR and LAI showing marked increases (Yu et al., 2025). During the maturity stage, both indicators further increased and exhibited strong spatial consistency, indicating that the crop had fully entered the physiological maturity stage, with canopy structure and light use efficiency approaching saturation. The spatiotemporal variations of the vegetation parameters FPAR and LAI provided key information for yield prediction and also validated the sensitivity and temporal phased responsiveness of remote sensing indicators to crop changes.

### 4.2 Differences in winter wheat yield prediction across growth stages and model behavior

Based on the performance comparison of the three models (**Table 2**), BP, RF, and PLS show clear differences in yield-prediction accuracy at different phenological stages, all achieving their best performance during the growth period, with BP performing the best (R²=0.81, RMSE=414.48). These differences are mainly due to the varying abilities of the models to capture relationships between variables and the different expression strengths of remote sensing and meteorological information across phenological stages.

From a temporal perspective, the R² values of the three models were generally low during the overwintering period. During this period, crops were in a dormant state under low temperatures, with relatively weak features from remote sensing and meteorological information and strong spatial consistency, which contributed little to yield prediction. In the maturity period, as crops entered the final stage of yield formation, the crop population became stable and the information entropy of variables was relatively low, leading to weaker predictive performance (Lou et al., 2024). By contrast, during the growth period, crops were in a phase of vigorous growth, the features reflected by remote sensing and meteorological information were of relatively high intensity, and spatial differences were pronounced, which effectively enhanced the predictive ability of the models and made this period the optimal time window for yield forecasting.

In terms of model performance, BP achieves higher prediction accuracy than RF and PLS across all phenological stages. The BP model can capture the complex coupled relationships among vegetation spectral, leaf area index, and meteorological variables through its multilayer nonlinear mapping structure, thereby improving yield-prediction accuracy (Ji et al., 2023). In comparison, although the RF model has some nonlinear modeling capability, it relies solely on training data for regression-tree splitting and lacks extrapolation ability beyond the training domain (Yu et al., 2024). This makes it difficult to handle agricultural conditions that fall outside the historical range, a limitation that may become critical when predicting crop yields under future scenarios. The PLS model, based on linear assumptions, has limited capacity to fit the complex processes of crop growth, resulting in overall lower prediction accuracy, which is consistent with the findings of Ma et al. regarding the limitations of linear models in biomass simulation (Ma et al., 2020).

### 4.3 SHAP visualization for interpreting the model

To further explore the predictive mechanism of the BP model during the growth period and assess the impact of each variable on winter wheat yield prediction, we applied SHAP visualization to interpret the “black-box model.”

**Figure 10:**
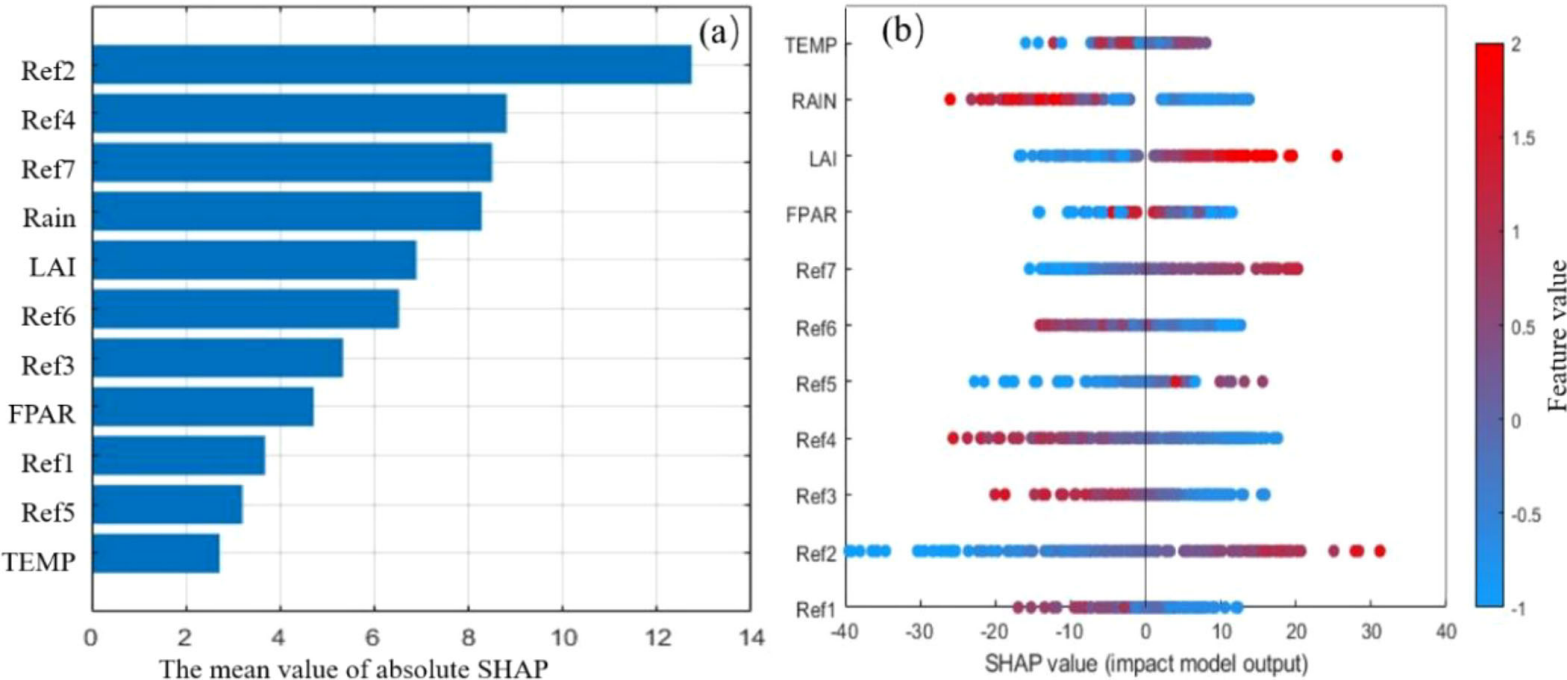
SHAP importance of multidimensional features. **(a)** Feature importance ranking; **(b)** SHAP value impact plot. Ref1–7 are multispectral reflectance, FPAR is Fraction of absorbed Photosynthetically Active Radiation, LAI is the leaf area index, RAIN is monthly precipitation, and TEMP is monthly mean temperature.

**Figure 10a** visualizes the mean absolute SHAP values of each feature in the winter wheat yield prediction model, reflecting the average contribution of each feature to the model’s prediction results (Srivastava et al., 2022). The results show that Ref2 has the highest mean absolute SHAP value among all features, with Ref4 and Ref7 also demonstrating relatively large influence. These reflectance bands may contain spectral information critical to winter wheat growth status and yield formation. RAIN and LAI, representing meteorological factors and vegetation indices, closely follow in the feature importance ranking and exert a significant impact on the model’s predictions. This highlights the crucial role of meteorological conditions and crop growth status in yield prediction. In addition to the top five important features described above, other features such as Ref6, FPAR, Ref1, Ref5, and TEMP, while relatively less important, still offer a certain amount of valuable information to the model.

**Figure 10b** presents the SHAP values of all features for each test sample, along with their ranges of influence. The color bar indicates the magnitude of feature values (red for high, blue for low), enabling us to observe how variations in feature values affect the prediction results. Focusing on the top five important features, Ref2, Ref7, and LAI exhibit clear trends: when these features have high values (red), their corresponding SHAP values are positive, indicating that higher feature values tend to increase the predicted yield (positive correlation); conversely, when their values are low (blue), the SHAP values are negative, suggesting that lower feature values tend to decrease the predicted yield (negative correlation). In contrast, Ref4 and RAIN show the opposite trend: high values correspond to negative SHAP values, potentially lowering the predicted yield, while low values correspond to positive SHAP values, potentially increasing the predicted yield. Additionally, the width of the colored regions in the SHAP values reflects the range of variation in a feature’s influence; the wider the region, the more pronounced the feature’s impact on the model’s predictions.

**Figure 11:**
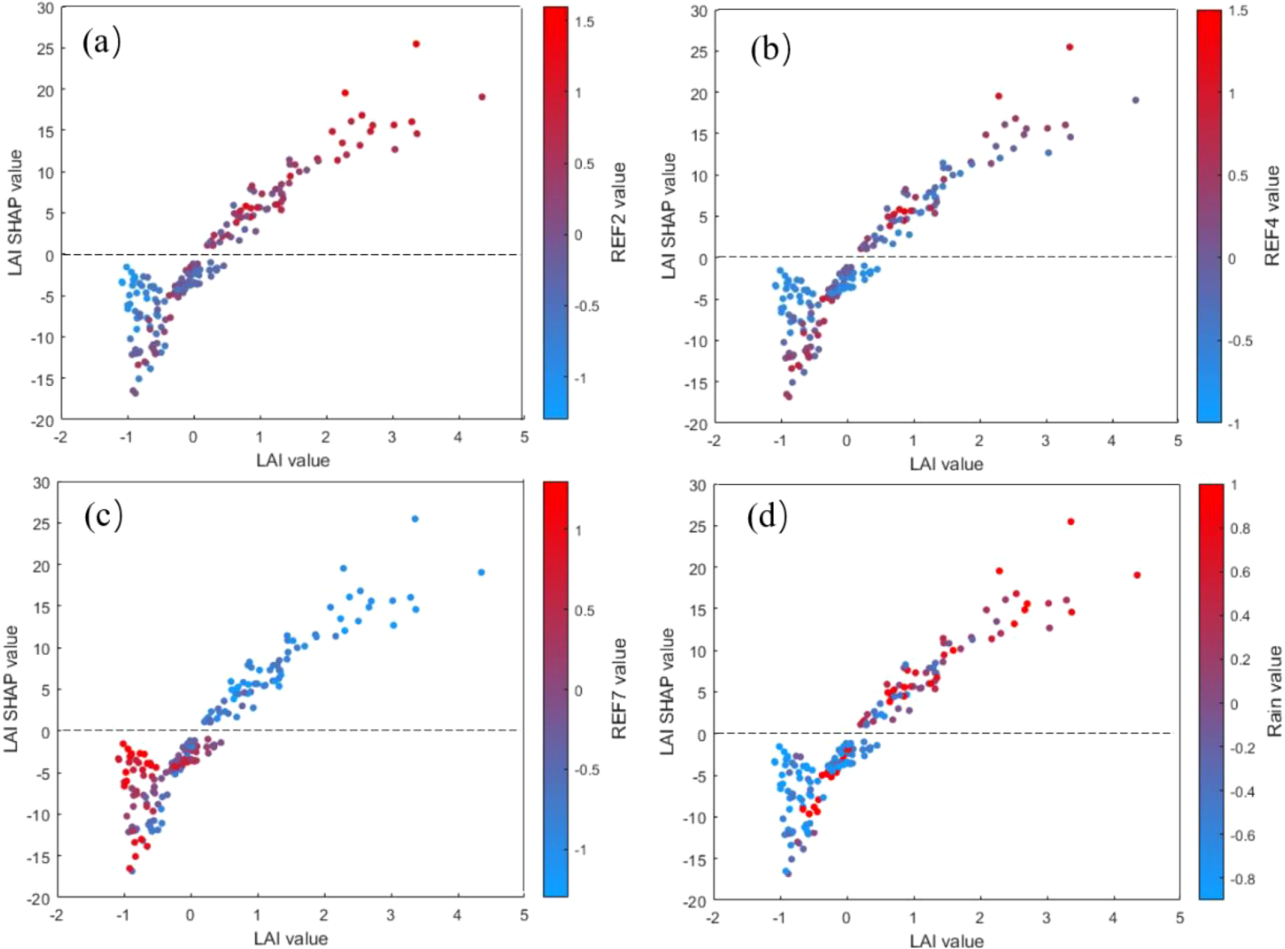
Dependence interactions between LAI and important features. **(a)** LAI vs. Ref2; **(b)** LAI vs. Ref4; **(c)** LAI vs. Ref7; **(d)** LAI vs. RAIN.

**Figure 11** shows the SHAP dependence plots for feature interactions, revealing how the contribution of one feature to the prediction changes with variations in another feature. To improve the interpretability and practicality of the model, this study examined the relationships of LAI feature values and LAI SHAP values with Ref2, Ref4, Ref7, and RAIN. Such interaction effects are more readily understood and accepted, helping to guide relevant strategies.

In **Figure 11a**, the color distribution of the sample points changes significantly from blue to red. When the LAI feature values are low, the Ref2 feature values are also low, and the LAI SHAP values are<0. Conversely, when the LAI feature values are high, the Ref2 feature values are also high, and the LAI SHAP values are >0. In **Figure 11d**, when the RAIN feature values are low, the LAI feature values are also low, and the LAI SHAP values are<0, indicating that this feature combination contributes negatively to yield prediction. In contrast, when the RAIN feature values are high, the LAI feature values are also high, and the LAI SHAP values are >0, suggesting that this feature combination contributes positively to yield prediction. In **Figure 11c**, the color distribution of the sample points changes significantly from red to blue. When the LAI feature values are low, the Ref7 feature values are high, and the LAI SHAP values are<0. Conversely, when the LAI feature values are high, the Ref7 feature values are low, and the LAI SHAP values are >0. In **Figure 11b**, however, the color distribution of the sample points is somewhat disordered: when the LAI feature values are either low or high, the Ref4 feature values vary widely, and the interaction effects are not apparent.

In summary, the SHAP analysis not only clarified the importance of each feature for the prediction of winter wheat yield by the BP model with the growth period, but also revealed the interactions among features and their influence on the prediction results. This indicates that the model can reasonably reflect the complex coupling effects of vegetation functional features, spectral features, and meteorological information, providing an intuitive basis for understanding yield formation mechanisms while enhancing the model’s interpretability and practical applicability.

## 5 Conclusion

This study demonstrates the potential of integrating MODIS remote sensing and meteorological data through county-level winter wheat yield prediction in the Huang–Huai–Hai region, while identifying the optimal periods (overwintering, growth, and maturity periods) and model for yield prediction. The results indicated that the growth period is the optimal window for predicting winter wheat yield, and the BP model consistently outperformed RF and PLS across multiple periods, achieving the highest accuracy during the growth period (R²=0.81, RMSE=414.48 kg/ha). In addition, SHAP value analysis revealed the influence of each feature in the model on yield prediction, with specific reflectance bands, precipitation, and LAI playing key roles in the model’s predictions. Using remote sensing and meteorological data from the 2023 growth period of winter wheat, combined with county-level yield data provided by the National Bureau of Statistics, the BP model was validated. The predicted results showed an R² of 0.73 and an RMSE of 509.30 kg/ha, further confirming the model’s accuracy and stability in practical winter wheat yield prediction. Although this study has achieved certain results in predicting winter wheat yield using the BP model during the growth period, the current research focuses only on data from a single phenological stage. This limits the model’s ability to capture the entire crop growth process. Therefore, the next stage of research will extend to the use of multi-period data, integrating information from the overwintering, growth, and maturity stages of winter wheat to construct an LSTM time-series model. The LSTM model can more effectively capture the growth dynamics of winter wheat throughout its entire cycle and more accurately simulate the yield formation process. Through this approach, we expect to achieve more precise and comprehensive winter wheat yield predictions, providing a scientific basis and methodological reference for regional agricultural monitoring and food security assurance.

## Data Availability

The original contributions presented in the study are included in the article/supplementary material. Further inquiries can be directed to the corresponding author.
